# Supplementary material for: Circadian RNA expression elicited by 3’-UTR IRAlu-paraspeckle associated elements
Source: eLife. 2016 Jul 21;5:e14837. doi: 10.7554/eLife.14837 (PMC4987140; doi:10.7554/eLife.14837)
Supplement: Figure 2—source data 1. — DOI: http://dx.doi.org/10.7554/eLife.14837.005 [file elife-14837-fig2-data1.docx]

**Figure 2- source data file 1**

**qPCR Primer list (rat) (5’ to 3’)**

Neat1-1 and 1-2

AAGGCACGAGTTAGCCGCAAAT

TGTGCACAGTCAGACCTGTCATTC

Neat1-2

GCCTGCTTTCAGCTGTTGGTTT

TCTGGACAGCAACTGAGCAATACG

Egfp

CGGCATCAAGGTGAACTTCAAGATCC

ACTGGGTGCTCAGGTAGTGGTT

Calr

ACCAGAAGGACATGCATGGAGACT

TTGTTGATCAGCACGTTCTTGCCC

Evi5

TGAGCACAGTGCAGCTTGTGAA

TGCTGACCATGAAGAGCTTACCGA

Maged1

GGCAGAACTTACGACCCTCACCTAAT

TGATGGGCACCTTCGTGTAGTCTT

Canx

TGGCAGCGACCTATGATTGACAAC

GAGCTCCAAACCAATAGCGCTGAA

Pcbp2

GGCGCAGATCAAAATTGCAAACCC

TGTCTCCGAGGAAAGCCTGACATT

Syne1

GCAGTCATTCTTGCTCCAACACCA

CGGCTGAACATCTCGGCTTGAAAA

Fkbp4

ACTTCCAAAAGGTCCTGCAGCTCT

CCACTTCTGTCTTGGCCTTATGTTCC

Gapdh

CCCTCAAGATTGTCAGCAATG

GTCCTCAGTGTAGCCCAGGAT

**qPCR Primer list (mouse) (5’ to 3’)**

Neat1-1 and 1-2

TGGGCCCTGGGTCATCTTACTAGATA

CACAGCTGTTCCAATGAGCGATCT

36B4 (Rplp0)

GCTGATGGGCAAGAACACCA

CCCAAAGCCTGGAAGAAGGA

**Antisens Oligonucleotides phosphorothioate**

CCACCATCATCAATCCTCTGG

AAGTCAGACCTGTCATTCAG

**Neat1 siRNA**

**Mix of 1/** GGAGGUCGACUUUGAACUUTT

AAGUUCAAAGUCGACCUCCTT

**2**/ GCAUUGAUCACGACUUUAUTT

AUAAAGUCGUGAUCAAUGCTT

**3**/ GCCUGUUGUUAAAUGGGUUTT

AACCCAUUUAACAACAGGCTT

**4**/ CCAACGUAGCCGUCUCUUUTT

AAAGAGACGGCUACGUUGGGT

**Negative control siRNA**

UUCUCCGAACGUGUCACGUTT

ACGUGACACGUUCGGAGAATT

**Neat1 RNA pull-down specific oligonucleotides (3’ biotinylated with a triethyleneglycol spacer)**

S oligo 1: CTCCACCATCATCAATCCTCTGGAC

S oligo 2: GCCTTCCCACATTTAAAAACACAAC

Non-specific: ATAATTTCAAACATCAAATGGTATTTTA

**Figure 2- source data file 1: Sequences of qPCR primers and oligonucleotides**
