# Supplementary material for: Circadian RNA expression elicited by 3’-UTR IRAlu-paraspeckle associated elements
Source: eLife. 2016 Jul 21;5:e14837. doi: 10.7554/eLife.14837 (PMC4987140; doi:10.7554/eLife.14837)
Supplement: Figure 3—source data 1. — DOI: http://dx.doi.org/10.7554/eLife.14837.007 [file elife-14837-fig3-data1.docx]

**Figure 3-source data file 1:**

|  | **Baseline** | **Amplitude** | **Phase-Shift** | **R^2^** |
| --- | --- | --- | --- | --- |
| **PSPC1** | 76.88 | 16.47 | 0.837 | 0.798 |
| **RBM14** | 65.24 | 20.57 | 0.00250 | 0.781 |
| **Neat1** | 63.5 | 25.08 | 1.142 | 0.616 |

**Figure 3-source data file 1:** **Cosinor analysis of the rhythmic expression pattern of paraspeckle components in GH4C1 cells**: PSPC1, RBM14 protein levels and Neat1 RNA levels displayed a rhythmic pattern in GH4C1 cells that could be fitted with a non-linear sine wave equation (Y = Baseline + Amplitude * sin (Frequency*X + Phase-shift) in which the period value (2pi/Frequency) was constrained to the circadian period value 24h. Presented are the best-fit values obtained with a R^2^>0.55.
