# Supplementary material for: Circadian RNA expression elicited by 3’-UTR IRAlu-paraspeckle associated elements
Source: eLife. 2016 Jul 21;5:e14837. doi: 10.7554/eLife.14837 (PMC4987140; doi:10.7554/eLife.14837)
Supplement: Figure 3—source data 2. — DOI: http://dx.doi.org/10.7554/eLife.14837.008 [file elife-14837-fig3-data2.docx]

**Figure 3-source data file 2:**

|  | **Baseline** | **Amplitude** | **Phase-shift** | **R^2^** |
| --- | --- | --- | --- | --- |
| **anti-NONO** | 256.2 | 172.8 | -0.911 | 0.736 |
| **anti-SFPQ** | 151.1 | 66.27 | -1.278 | 0.56 |
| **anti-RBM14** | 129.7 | 37.86 | -10.92 | 0.811 |
| **anti-PSPC1** | 265.7 | 145.4 | -0.641 | 0.653 |

**Figure 3-source data file 2:** **Cosinor analysis of the rhythmic binding of the four paraspeckle-associated proteins on Neat1 RNA in GH4C1 cells :** binding of NONO, SFPQ, PSPC1 and RBM14 on Neat1 RNA displayed a rhythmic pattern in GH4C1 cells that could be fitted with a non-linear sine wave equation (Y = Baseline + Amplitude * sin (Frequency*X + Phase-shift) in which the period value (2pi/Frequency) was constrained to the circadian period value 24h. Presented are the best-fit values obtained with a R^2^>0.55.
