# Supplementary material for: Circadian RNA expression elicited by 3’-UTR IRAlu-paraspeckle associated elements
Source: eLife. 2016 Jul 21;5:e14837. doi: 10.7554/eLife.14837 (PMC4987140; doi:10.7554/eLife.14837)
Supplement: Figure 5—source data 1. — DOI: http://dx.doi.org/10.7554/eLife.14837.014 [file elife-14837-fig5-data1.docx]

**Figure 5-source data file 1:**

|  | **Baseline** | **Amplitude** | **Phase-shift** | **R^2^** |
| --- | --- | --- | --- | --- |
| **Alu-egfp cell line** | 61.31 | 34.2 | -1.791 | 0.726 |
| **IRAlu-egfp cell line** | 257.5 | 180.2 | -1.236 | 0.686 |

**Figure 5-source data file 1:** **Cosinor analysis of the rhythmic ratio of nuclear versus cytoplasmic egfp mRNA levels in Alu-egfp and IRAlu-egfp cell lines:** nuclear versus cytoplasmic egfp mRNA levels displayed a rhythmic pattern in Alu-egfp and IRAlu-egfp cells that could be fitted with a non-linear sine wave equation (Y = Baseline + Amplitude * sin (Frequency*X + Phase-shift) in which the period value (2pi/Frequency) was constrained to the circadian period value 24h. Presented are the best-fit values obtained with a R^2^>0.55.
