# Supplementary material for: Circadian RNA expression elicited by 3’-UTR IRAlu-paraspeckle associated elements
Source: eLife. 2016 Jul 21;5:e14837. doi: 10.7554/eLife.14837 (PMC4987140; doi:10.7554/eLife.14837)
Supplement: Figure 6—source data 2. — DOI: http://dx.doi.org/10.7554/eLife.14837.019 [file elife-14837-fig6-data2.docx]

**Figure 6-source data file 2:**

|  | **Baseline** | **Amplitude** | **Phase-shift** | **R^2^** |
| --- | --- | --- | --- | --- |
| **Calr** | 203.9 | -100.8 | 0.954 | 0.773 |
| **Canx** | 48.65 | 27.88 | 10.51 | 0.557 |
| **Fkbp4** | 140.3 | 78.10 | -0.839 | 0.592 |
| **Evi5** | 105.2 | 39.71 | 1.465 | 0.828 |
| **Maged1** | 215.7 | 107.1 | -0.5327 | 0.578 |
| **Pcbp2** | 43.99 | 17.26 | -0.5104 | 0.592 |
| **Syne1** | 319.7 | 137.6 | -0.6961 | 0.552 |

**Figure 6-source data file 2:** **Cosinor analysis of the relative ratio nuclear/cytoplasmic mRNA of seven Neat1 RNA targets in GH4C1 cells :** ratio levels of the seven Neat1 RNA targets displayed a rhythmic pattern in GH4C1 cells that could be fitted with a non-linear sine wave equation (Y = Baseline + Amplitude * sin (Frequency*X + Phase-shift) in which the period value (2pi/Frequency) was constrained to the circadian period value. Presented are the best-fit values obtained with a R^2^>0.55.
